# Supplementary material for: Viral Inactivation by Light-Emitting Diodes: Action Spectra Reveal Genomic Damage as the Primary Mechanism
Source: Viruses. 2025 Jul 30;17(8):1065. doi: 10.3390/v17081065 (PMC12390714; doi:10.3390/v17081065)
Supplement: Supplementary file 1 [file viruses-17-01065-s001.zip › viruses-3699813-supplementary.pdf]

Supplementary Materials

Viral Inactivation by Light-Emitting Diodes: Action Spectra Reveal Genomic Damage as the Primary Mechanism

Table S1. Peak wavelength and fluence rate of the light-emitting diodes (LEDs) used in this study.

LED wavelength	Peak wavelength (nm) – measured	Fluence rate (mW/cm^2)
U250	250.8	0.5
U254	253.3	1.0
U257	255,7	1.0
U260	260.3	1.0
U263	263.0	1.0
U267	266.8	1.0
U270	269.3	1.0
U275	274.0	1.0
U280	281.3	1.0
U290	289.7	1.5
U300	300.2	1.0
U308	307.5	1.0
U365	367.0	18.0

Table S2. Primers for strand-specific RT-qPCR.

Gene	RT sequence (5′–3′)	qPCR forward sequence (5′–3′)	qPCR reverse sequence (5′–3′)
	Influenza A virus strain A/human/Puerto Rico/8/1934 (H1N1)		
NA	ggccgtcatggtggcgaat	ggccgtcatggtggcgaat	acatcactttgccggtatcagggt
	Severe acute respiratory syndrome coronavirus 2/Hu/DP/Kng/19-020		
N	tcccctactgctgcctggag	tctgataatggaccccaaaat	tcttccttgccatgttgagtgag
	Human coronavirus-OC43		
N	ttcttccaattggccataatt	cgttctggtaatggcatcct	tcctttccctttgaaactg
	Respiratory syncytial virus strain long		
F	tacctcaacggttaggagtttc	aggtcctgcacttagaaggaga	ttctgcagctttgcttattcaca
	Human metapneumovirus strain TN/83-1211		
F	gtcttcctgtgctgactttg	atggctgtcagcttcagtca	atcctgcagatgttggcatg

NA, neuraminidase gene; *N*, nucleocapsid gene; *F*, fusion glycoprotein gene.

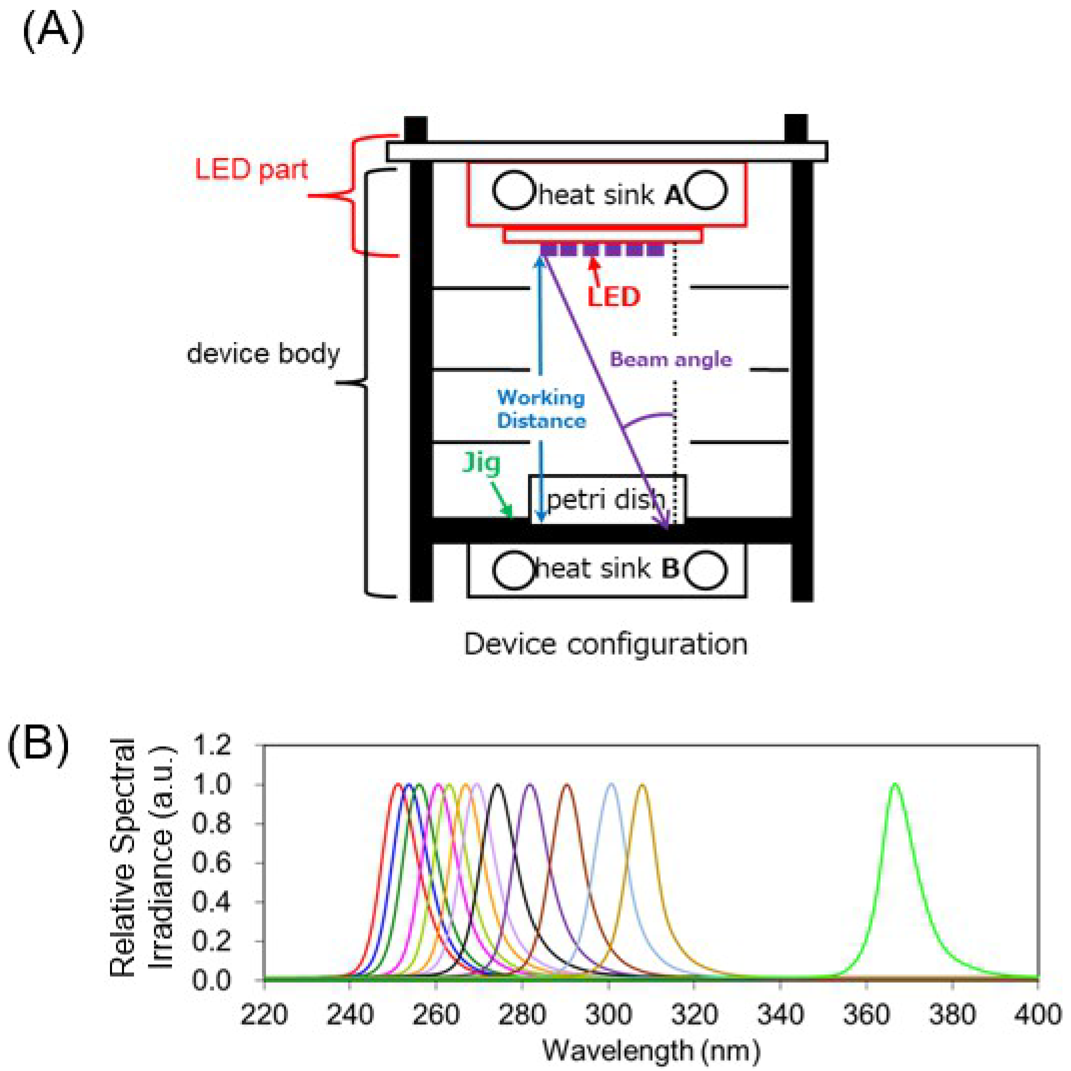

Figure S1. Schematic of the system equipped with interchangeable light-emitting diodes (LEDs) at 13 different wavelengths. (A) System configuration. (B) Relative spectral irradiance of the LEDs.

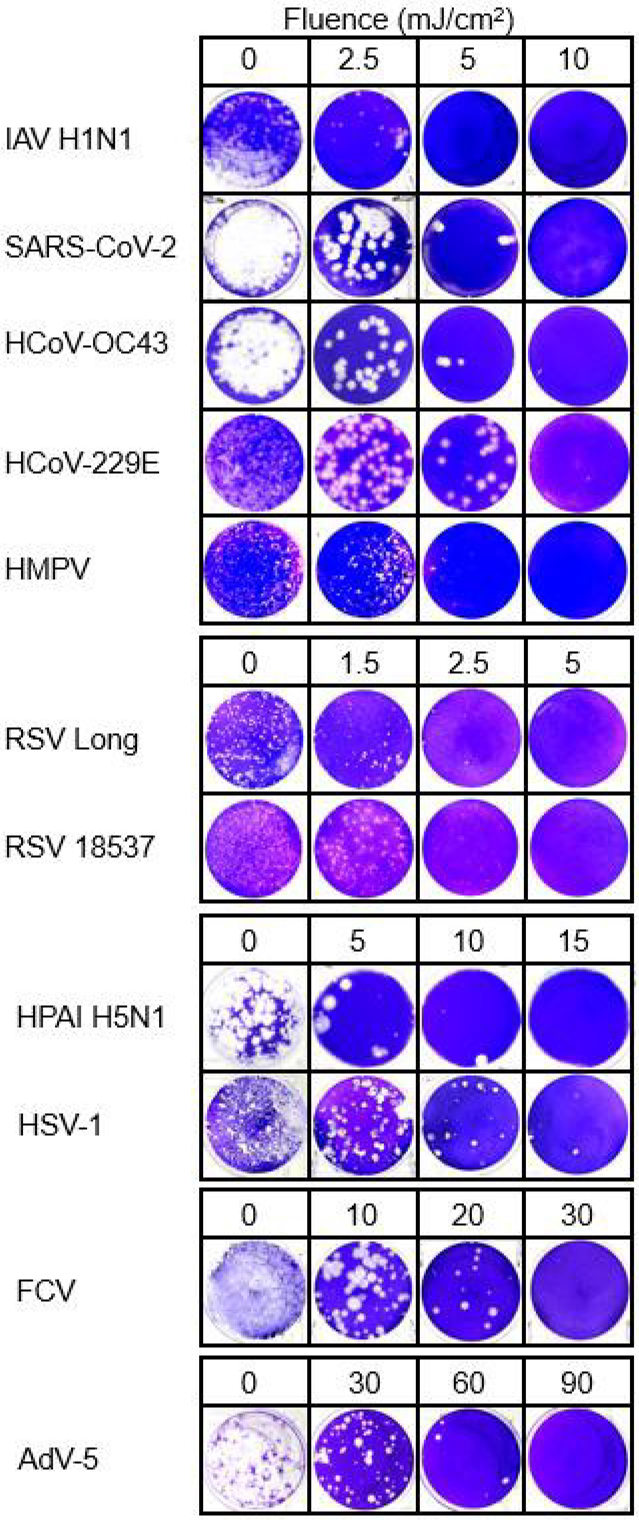

Figure S2. Related to Figure 1. Reduction in viral infectivity induced by irradiation with U280 light-emitting diodes (LEDs). Representative images of plaque-forming assays used to measure the virucidal effect of U280-LED irradiation. Viral suspensions were irradiated with U280-LEDs at the indicated fluences and then used to infect the host cells. Viral infectivity was quantified by the PFU assay. IAV, influenza A virus; HPAI, highly pathogenic avian influenza virus; SARS-CoV-2, severe acute respiratory syndrome coronavirus 2; HCoV, human coronavirus; RSV, respiratory syncytial virus; HMPV, human metapneumovirus; HSV-1, herpes simplex virus type 1; FCV, feline calicivirus; AdV-5, adenovirus type 5.

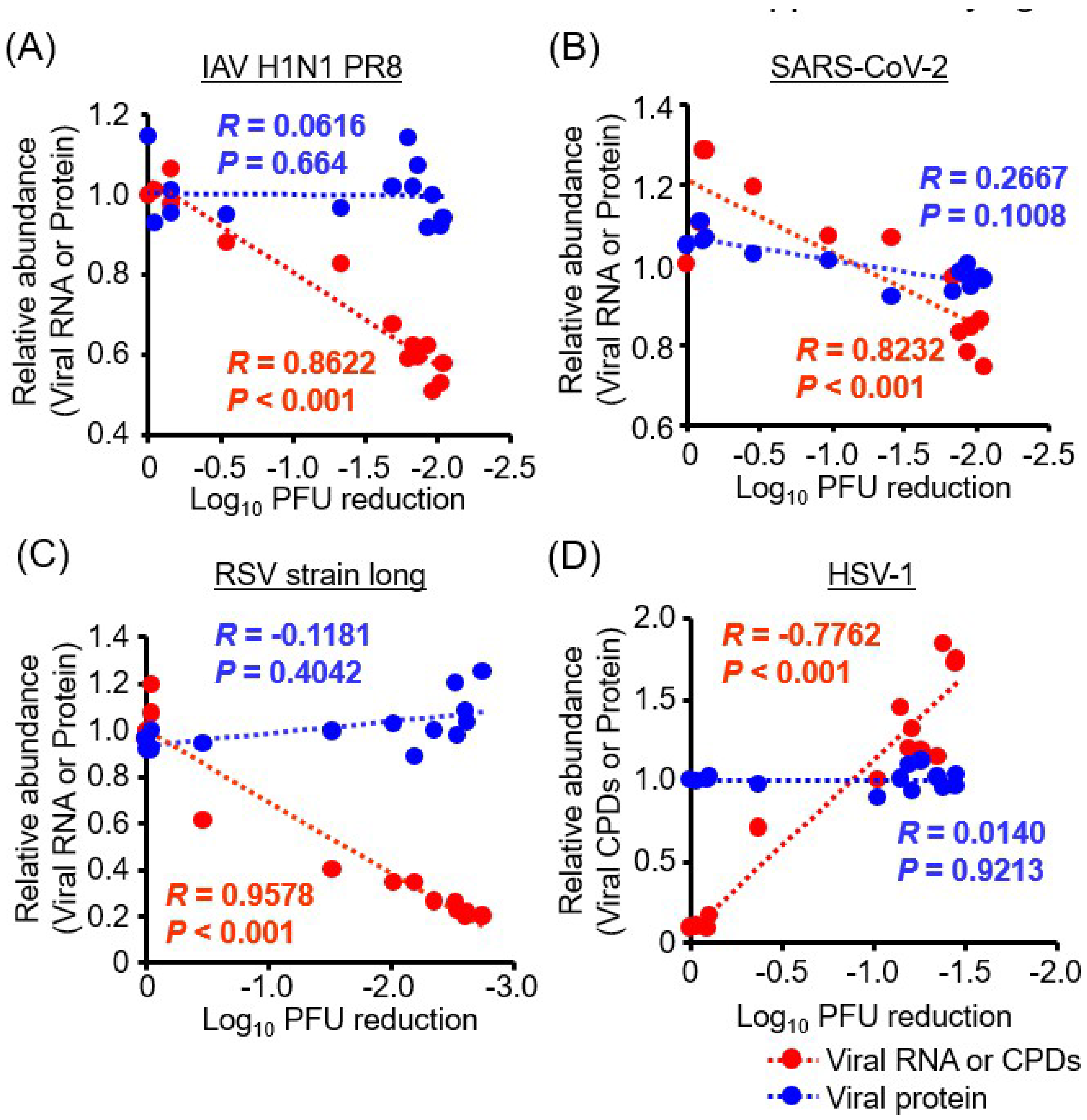

Figure S3. Relationship between the ultraviolet (UV) action spectra of light-emitting diodes (LEDs) for infectivity reduction and damage to the viral genome or protein. (A) Influenza A virus (IAV) strain A/human/Puerto Rico/8/1934 (H1N1). (B) Severe acute respiratory syndrome coronavirus 2 (SARS-CoV-2) Hu/DP/Kng/19-020. (C) Respiratory syncytial virus (RSV) strain long. (D) Herpes simplex virus-1 (HSV-1) strain KOS. Each plot presents the infectivity reduction (as indicated in Figure 2) and degradation of viral genomic RNA (Figure 3), DNA, or protein (Figure 7) following irradiation at each LED wavelength. Spearman's rank correlation test was used to analyze the association between two variables. Spearman's correlation coefficient (*r*) was transformed into *z* scores using Fisher's *r*-to-*z* transformation and assessed for statistical significance by calculating the observed *z* test statistic. Results are displayed as the mean ± SD.
